# A pilot study for assessing NAO humanoid robot assistance in shoulder rehabilitation

**DOI:** 10.1002/jeo2.70122

**Published:** 2024-12-30

**Authors:** Alessandra Raso, Martina Pulcinelli, Emiliano Schena, Alfio Puglisi, Giovanni Pioggia, Arianna Carnevale, Umile Giuseppe Longo

**Affiliations:** ^1^ Fondazione Policlinico Universitario Campus Bio‐Medico Roma Italy; ^2^ Department of Engineering, Laboratory of Measurement and Biomedical Instrumentation Università Campus Bio‐Medico di Roma Rome Italy; ^3^ Institute for Biomedical Research and Innovation (IRIB) National Research Council of Italy (CNR) Messina Italy; ^4^ Research Unit of Orthopaedic and Trauma Surgery, Department of Medicine and Surgery Università Campus Bio‐Medico di Roma Roma Italy

**Keywords:** adherence to rehabilitation programmes, orthopaedics, robot‐assisted upper limb rehabilitation, shoulder rehabilitation, upper limb rehabilitation

## Abstract

**Purpose:**

This study aimed to explore the potential application of NAO in guiding patients through rehabilitative exercises using external audiovisual stimuli, focusing on temporospatial control in terms of range of motion (ROM), execution time and movement smoothness.

**Methods:**

This is a preliminary analysis involving ten healthy volunteers and two patients with shoulder musculoskeletal disorders. The protocol was developed in two phases (III and IV) with different ROM limits and including flexion–extension (FE), external‐rotation (ER) and internal‐rotation (IR) exercises, performed at two speeds and both with and without NAO assistance. Simultaneously, upper limb kinematics were assessed using a stereophotogrammetric system as a reference. Performance was evaluated by mean absolute error (MAE) for ROM and execution time, with smoothness assessed through Log Dimensionless Jerk analysis.

**Results:**

In phase III, results for volunteers showed ROM differences in FE and ER, while IR was unaffected by NAO presence. In phase IV, NAO assistance resulted in reduced MAE across nearly all exercises. Patients who only performed phase III exercises at lower speed stayed within ROM limits for all movements performed with NAO, except for ER. For all the participants, results showed a significant reduction in the time MAE when using NAO. Patients exhibit greater smoothness during FE performed with NAO.

**Conclusions:**

NAO showed potential in aiding patients with shoulder musculoskeletal disorders to replicate rehabilitation exercises, guiding both ROM and timing while influencing movement smoothness. NAO imitation could lead to improved rehabilitation outcomes and enhanced motor learning of motor skills, fostering greater adherence to prescribed therapy.

**Level of Evidence:**

Level V, diagnostic.

## Graphical abstract

Humanoid robot‐assisted rehabilitation for shoulder musculoskeletal disorders: evaluation of the assistance in terms of range of motion, execution time, and smoothness of movement.

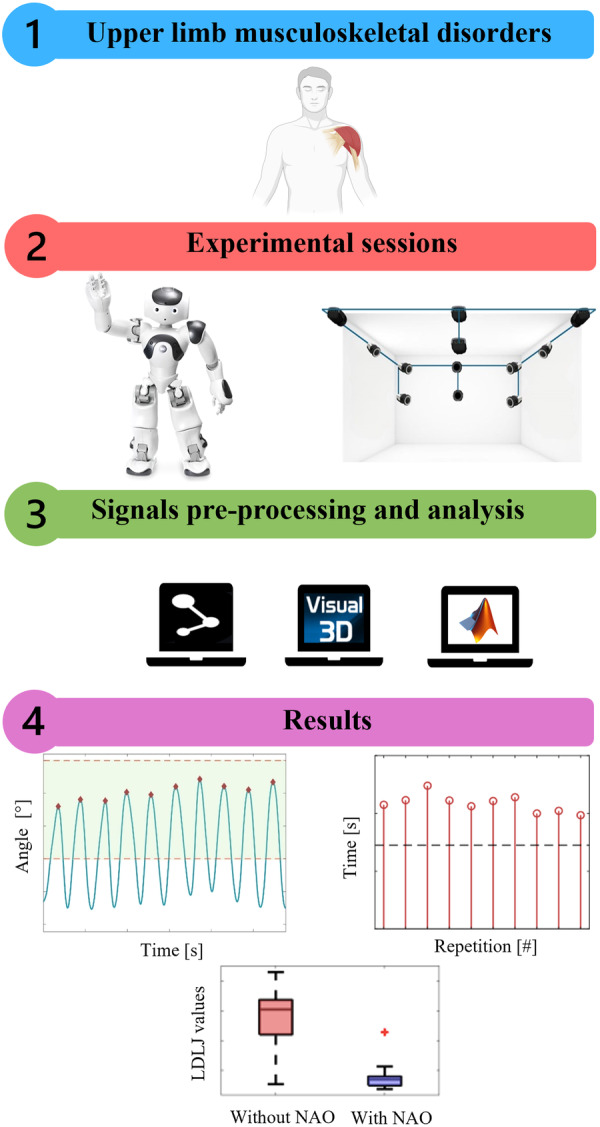

**Abbreviations**
DLJ: dimensionless jerk
ER: external rotation
FE: flexion–extension
IR: internal rotation
LDLJ: log dimensionless jerk
MAE: mean absolute error
ROM: range of motion
SARs: socially assistive robots

## INTRODUCTION

The shoulder joint is known for being the most mobile but also the most unstable joint in the human body due to its complexity, making it susceptible to various injuries and conditions, such as rotator cuff tears [7, 16]. Specifically, as reported by the American Academy of Orthopaedic Surgeons, rotator cuff tears stand out as a prevalent aetiological factor in adult shoulder pain, encompassing a substantial 85% of reported cases [17]. Treatments can be broadly categorized into nonsurgical management and surgical repair, with the choice depending on several clinical factors such as age, type of injury and tear size [8, 12].

Rehabilitation is essential for restoring motor functions after surgery [2, 11, 25], with protocols usually progressing from passive to active and strengthening exercises, aiming to recover limb functionality and mobility [2, 4, 25].

Robotics has emerged as a valuable tool in rehabilitation, offering consistent, repeatable movements and enhanced functional recovery [9, 10, 27]. Socially assistive robots (SARs) have gained attention for their ability to engage patients, particularly in contexts like autism treatment, and have been explored as aids in rehabilitation exercises [9, 14, 19]. These robots can improve patient adherence to rehabilitation programmes, enhance motivation and support physiotherapists [15].

Amongst the various SARs, NAO, developed by Aldebaran Robotics, stands out due to its successful application in education and rehabilitation [3, 6]. Major clinical studies have primarily focused on the use of NAO to assess patient engagement, demonstrating its effectiveness in rehabilitation, and facilitating socialization amongst patients with motor limitations [29, 30]. The primary applications have been in the rehabilitation of children with autism spectrum disorder and in clinical contexts, such as speech disorders and Down syndrome [9, 20, 30]. Preliminary results have indicated that patients respond positively to the system, showing active engagement during therapy sessions.

The objective of this study was to explore the potential application of NAO in guiding patients with shoulder musculoskeletal disorders through rehabilitative exercises using external audiovisual stimuli, with a focus on temporospatial parameters—namely, range of motion (ROM) and execution time—as well as movement smoothness.

## METHODS

Ten healthy volunteers and two patients were enroled in the present study at 6 months postintervention follow‐up. Before experimental sessions, all subjects read and signed an informed consent, approved by the Ethical Committee of University Campus Bio‐Medico of Rome (protocol code: 15.1(21).21 OSS ComEt UCBM).

### Rehabilitation protocol

The exercises and rehabilitation phases have been defined following ‘The American Society of Shoulder and Elbow Therapists’. Specifically, the last two phases of the rehabilitation process, phase III and phase IV, were selected for their focus on active patient movements that can be optimally adapted to the capabilities and functions of the NAO robot.

The following exercises were selected for each phase:

- Flexion–extension (FE): This exercise involves moving the arm forward (flexion) and then returning it to the neutral position (extension).
- External rotation (ER): In this exercise, the arm is rotated outward away from the body while keeping the elbow bent at a 90° angle.
- Internal rotation (IR): This exercise involves rotating the arm inward towards the body, again with the elbow bent at 90°.

The ROM criteria for phase III were set between 130° and 155° for FE, between 30° and 45° for ER and between 20° and 50° for IR. In phase IV, the ROM targets were increased to greater than or equal to 140° for FE, greater than or equal to 45° for ER and greater than or equal to 50° for IR. A summary of this information is given in Table 1.

**Table 1:** ROM specified by the ASSET protocol for each exercise and phase.

	ROM	
Exercise	Phase III	Phase IV
FE	130°≤ROM≤155°	ROM≥140°
ER	30°≤ROM≤45°	ROM≥45°
IR	20°≤ROM≤50°	ROM≥50°

Abbreviations: ASSET, The American Society of Shoulder and Elbow Therapists; ER, external rotation; FE, flexion–extension; IR, Internal rotation; ROM, range of motion.

All the exercises are implemented in NAO at two different speeds, a slow one of 30°/s (S_1_) and a fast one of 50°/s (S_2_). In particular, the aim was to set two speeds that could represent different patient needs while avoiding potential risks, considering that the optimal execution speed can vary greatly depending on the level of recovery and individual motor skills [18, 24].

Both healthy volunteers and patients enroled in this study underwent two sessions: the first with the robot and the second without its aid. Healthy volunteers performed the exercises of the two phases (III and IV) at both speeds (S_1_ and S_2_) with the robot, and then without the robot. Patients performed the exercises of one phase (III or IV) at one speed (S_1_ or S_2_), depending on their condition, with the robot, and then without the robot. This approach allowed a direct comparison of the parameters describing the quality of movement in assisted and unassisted exercises.

#### Protocol performed by healthy volunteers

Healthy volunteers performed FE, ER and IR in both phase III and phase IV at both speeds. Each movement was repeated 10 times, providing a robust set of data for each exercise.

**Figure 1:**
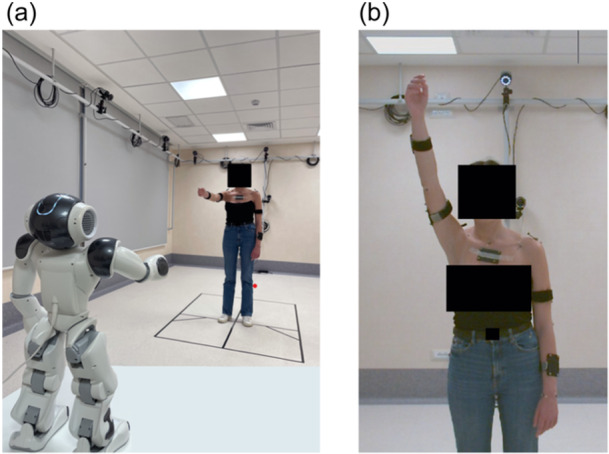
A healthy volunteer performing the exercises with the assistance of the NAO robot (a) and without robot assistance (b).

As already mentioned, the exercises were carried out in two sessions: first, the volunteers performed the exercises with the assistance of the NAO robot (see Figure 1a); second, without the assistance of the NAO robot, using a protractor to give the volunteers rough guidance on how to perform the movements (see Figure 1b). This dual approach allowed for a direct comparison between assisted and unassisted exercises.

#### Protocol performed by patients

Two patients who were at 6 months into their rehabilitation follow‐up were selectively enroled. These patients were allocated to phase III to align with the progression of their therapy. Each patient was assigned only one of the two predetermined exercise speeds, that is, S_1_, based on their individual condition and therapist's evaluation. Patients performed the designated exercises twice. The first time with NAO assistance, where the robot took the lead, demonstrating the exercises and setting the pace. Meanwhile, a therapist monitored the patient's performance, ensuring that the movements were executed safely. Subsequently, the same exercises were conducted without NAO's assistance, during which a therapist was present to guide the patient through the therapy, similar to a standard rehabilitation session. This approach mirrored the real‐life scenario where a therapist instructs and corrects the patient's movements, ensuring the exercises are carried out accurately and safely.

### NAO programming

The NAO robot was programmed using Choregraphe in the Python environment. Choregraphe allows for creating complex behaviours using a series of basic behaviour blocks, making it a user‐friendly interface for robot programming [29]. Each block represents a specific task for NAO, and combining these blocks enables the generation of new behaviours. In the context of rehabilitation therapy, four distinct configurations were implemented in Choregraphe: phase III at speed S_1_, phase III at speed S_2_, phase IV at speed S_1_ and phase IV at speed S_2_. The flexible programming of NAO in Choregraphe thus allows for the therapy to be adapted to a wide range of patients, enhancing the effectiveness of the rehabilitative treatment.

**Figure 2:**
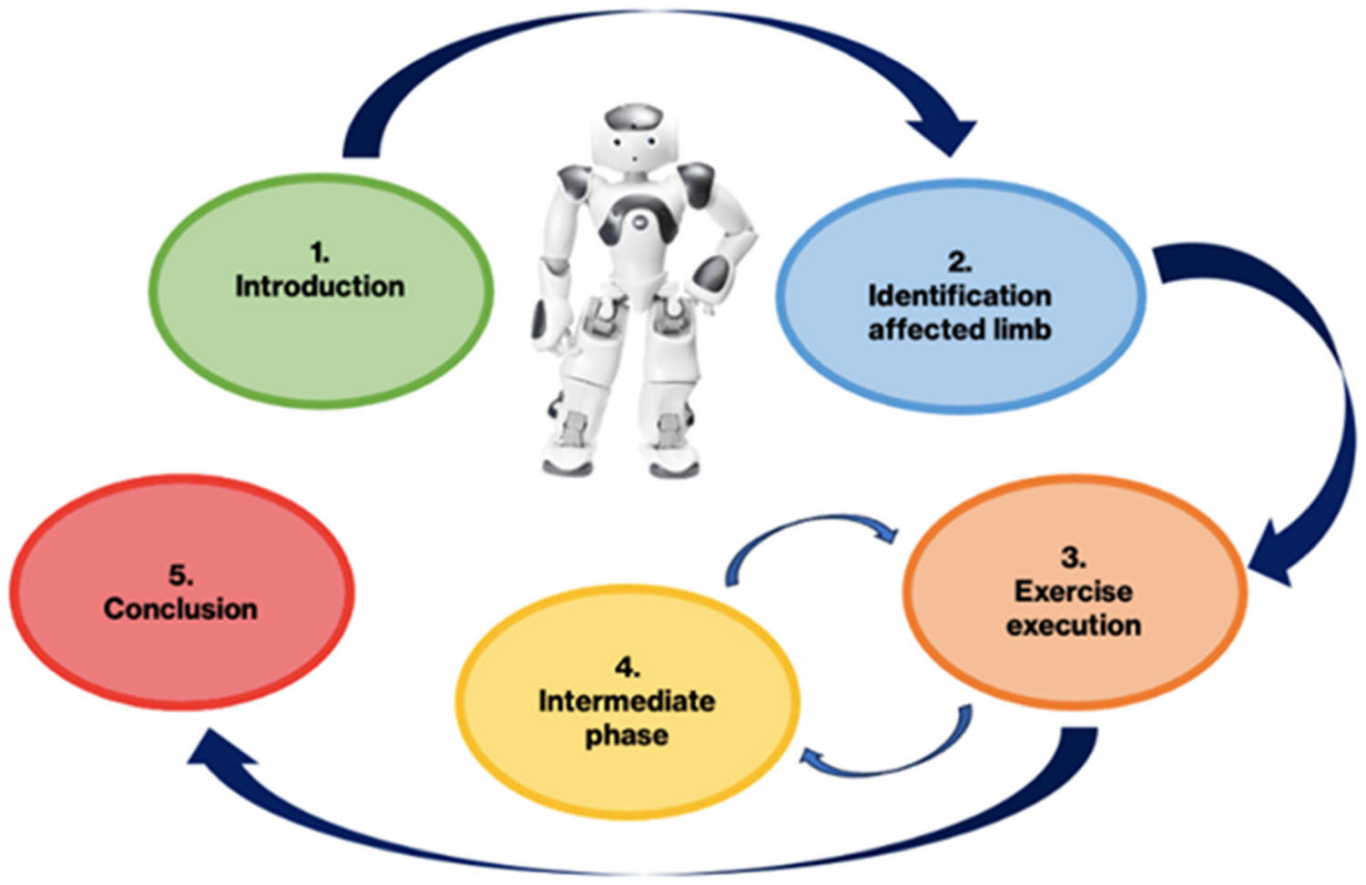
Loop of protocol implementation.

NAO's programming leverages a series of ‘boxes’ to create a fluid and adaptable interaction with the patient. As outlined below, the implementation of the therapy can be segmented into distinct phases: Introductory Phase, Identification of the Pathological Limb, Rehabilitative Therapy Exercises and Conclusion of the Rehabilitative Session. Additionally, situated between the exercises are the Intermediate Phases (see Figure 2).

1. *Introduction*: NAO asks the participant if they are ready to begin the rehabilitation treatment, awaiting a response. When it recognizes an affirmative response, it proceeds to the next phase.
2. *Identification of the affected limb*: NAO asks the subject if the compromised limb is the right or left one. When it recognizes a response of ‘right’ or ‘left’, it proceeds to the next phase.
3. *Exercise execution*: In this phase, NAO performs three actions. First, it provides a brief vocal description of the exercise. Second, NAO demonstrates two repetitions of the exercise for illustrative purposes. Finally, it guides the participant in performing the exercise, leading them through a series of ten repetitions at the speed and ROM defined by the protocol. At the end of the ten repetitions, it moves on to the next phase.
4. *Intermediate phase between exercises*: NAO asks the subject if they are ready to continue the session with a new exercise. When it recognizes an affirmative response, it proceeds to a phase identical to the previous one (phase #3), with the only difference being the assigned movement. This step is repeated three times (for each of the three exercises specified by the protocol). At the end of the third iteration, it proceeds to phase #5.
5. *Conclusion*: NAO thanks and bids farewell to the participant.

### Data collection and analysis

Shoulder kinematics were recorded using the Qualisys™ motion capture system (Qualisys AB) as a gold standard. In each experimental session, photoreflective markers (each with a diameter of 8 mm) were meticulously placed on specific anatomical landmarks. These included the jugular notch, xiphoid process, C7 vertebra and T8 vertebra, as well as bilateral placement on the acromioclavicular joint, coracoid process, medial and lateral epicondyles, radial and ulnar styloid [28]. Additionally, five rectangular‐shaped clusters, each containing four markers, were affixed to the thorax and bilaterally to the upper arms and forearms. The trajectories of these markers were recorded by 10 Miqus M3 cameras at a sampling frequency of 100 Hz, collected, and preprocessed using Qualisys Track Manager software (e.g., gap filling, labelling). Subsequently, these were imported into Visual 3D (C‐Motion Inc.) for kinematic analysis through a custom pipeline. In Visual 3D, trajectories of anatomical markers during a static N‐pose (held for 3 s) were utilized to define the local coordinate system and orientation of the thorax, humerus, and forearm [28]. The humerus 3D orientation was expressed relative to the thorax. This methodology was applied to analyse humerothoracic angles such as FE, IR and ER [5]. Finally, the kinematic results obtained in Visual 3D were analysed in MATLAB®. Three aspects describing the quality of movement were analysed, namely ROM, execution time and smoothness.

**Figure 3:**
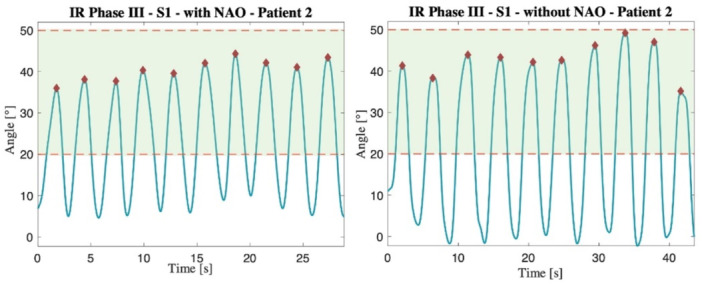
Waveforms of the IR exercise performed by patient 2 (phase III at S1 speed), both with and without NAO. In red are displayed the peak values, which are representative of the ROM achieved, and in green, the range defined by the clinical protocol. IR, Internal rotation; ROM, range of motion.

The waveforms of FE, ER and IR for each volunteer and patient were processed, and the peak values achieved in each repetition were identified (Figure 3). This evaluation was applied to all exercises performed by the subjects, both with the use of NAO and without.

For each identified repetition, it was checked whether the corresponding peak value satisfied the set ROM condition. The error committed for the lower limit and the upper limit were calculated through the difference between the lower and the upper values of the ROM specified by the clinical protocol, $x_{\mathrm{GS}}$, and the value $x_{i}$ achieved for the *i*th repetition by the *j*th subject. Subsequentially, the mean absolute error (MAE) for each exercise (FE, ER and IR) performed by the subjects was calculated for both upper and lower limits as shown below:

(1) $${\mathrm{MAE}}_{j}=\frac{1}{n}\sum_{i=1}^{n}|x_{\mathrm{GS}}-x_{i}|,$$

where *j* represents the *j*th subject, and *n* the number of repetitions.

This calculation was performed for both the lower and the upper limits in phase III, and only for the lower limit in phase IV.

To enable a comparison between the MAEs achieved with the assistance of NAO and those attained without it, the MAEs of all the subjects obtained for the FE, ER and IR exercises and considering both upper and lower limits were averaged:

(2) $${\mathrm{MAE}}_{\mathrm{overall}}=\frac{1}{N}\sum_{j=1}^{N}{\mathrm{MAE}}_{j},$$

where *N* represents all the subjects.

**Figure 4:**
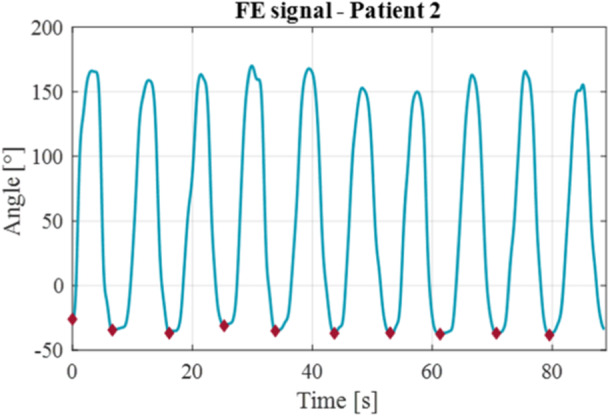
Graph illustrating the minimum peaks in the FE signal for patient two in phase III, performed at S_1_. Each red mark indicates the location of a minimum peak in the signal. FE, flexion‐extension.

The agreement between the assigned and effective execution time was calculated by quantifying the MAE of the duration of all the repetitions for each exercise. Initially, the time taken by each subject to complete the *i*th repetition was determined. This was done by identifying the minimum peaks of the signal, which allowed distinguishing individual repetitions (Figure 4).

Through the identification of the minimum peaks, the vector $t_{\mathrm{rip}}$ was created, which comprises the times for each repetition for the *j*th subject. For the repetitions preceding the tenth, the vector extends to just below the next index, while for the last repetition, it extends to the last index.

Next, the error was calculated by comparing the time defined as the benchmark ($t_{\mathrm{GS}}$, varying according to the speed of S1 or S2 exercises) with each value of the vector of times already determined. For the *j*th subject, the MAE was calculated as follows:

(3) $${\mathrm{MAE}}_{j}=\frac{1}{n}\sum_{i=1}^{n}|t_{\mathrm{GS}}-t_{\mathrm{rip},i}|,$$

where $t_{\mathrm{rip},i}$ is the time of the *i*th repetition, and n is the total number of repetitions.

Finally, the overall MAE was determined for all subjects involved in the study:

(4) $${\mathrm{MAE}}_{\mathrm{overall}}=\frac{1}{N}\sum_{j=1}^{N}{\mathrm{MAE}}_{j},$$

where *N* represents all the subjects.

The smoothness of movements was evaluated using the log dimensionless jerk (LDLJ), a jerk‐based metric that quantifies changes in acceleration. Smoother movements result in a lower LDLJ value. This metric, differing from the dimensionless jerk (DLJ) in its use of a logarithmic scale, facilitates the analysis of movements with large variations in jerk, making it particularly useful in clinical and rehabilitation settings [23]. However, these metrics are not suitable for evaluating entire rhythmic movements, as these would be interpreted as long discrete movements. Therefore, signal segmentation, a technique that divides a movement into several discrete parts, was performed. Specifically, each repetition of the FE exercise was divided into an ascending and a descending phase, while the extrarotation and intrarotation were divided into a rotation phase and a return to the initial position. Once the jerk of the discrete parts was calculated, the global one was obtained through their concatenation and the LDLJ was calculated as follows [1]:

(5) $$\mathrm{DLJ}≜-\frac{\left(t_{2}-t_{1}\right)}{v_{\mathrm{peak}}^{2}}\int_{t_{1}}^{t_{2}}{\left|\frac{d^{2}v\left(t\right)}{dt^{2}}\right|}^{2},$$

(6) LDLJ≜−ln|DLJ|,

where v(t) is the movement speed, $t_{1}$ and $t_{2}$ are the start and end times of the movement and $v_{\mathrm{peak}}^{2}$ is the peak speed.

## RESULTS

### ROM analysis

In accordance with previous instructions, the MAE was determined by analysing the disparity between the guidelines established in the rehabilitation protocol, considered the gold standard, and the actual ROM achieved by the participants. This approach was systematically applied to both the lower and upper bounds across all tasks executed by the subjects. Specifically, in the context of Phase IV, which was exclusively undertaken by volunteers, only the MAE pertaining to the lower limit was assessed. This is attributed to the absence of an upper limit specification within the protocol for this phase. Generally, a zero MAE value indicates that the ROM achieved by the subjects was within the limits set by the clinical protocol. The comprehensive results of the MAE of the volunteers are displayed in Table 2.

**Table 2:** MAEs of ROM related to the attainment of the lower and upper FE, ER and IR limits for phase III S1 and S2 and phase IV S1 and S2 of the volunteers.

Speed	Exercise	Phase III				Phase IV	
		Inferior limit		Superior limit		Inferior limit	
		MAE (°) with NAO	MAE (°) without NAO	MAE (°) with NAO	MAE (°) without NAO	MAE (°) with NAO	MAE (°) without NAO
S_1_	FE	10.7	0.4	1.3	6.9	1.5	0.0
S_2_	FE	3.2	0.4	3.3	5.5	0.0	3.9
S_1_	ER	16.4	22.3	0.0	0.0	33.3	34.4
S_2_	ER	27.2	28.4	0.0	0.0	43.4	41.8
S_1_	IR	4.0	4.5	0.1	0.0	16.5	16.8
S_2_	IR	5.1	3.8	0.0	0.0	26.0	26.9

Abbreviations: ER, external rotation; FE, flexion–extension; IR, Internal rotation; MAE, mean absolute error; ROM, range of motion.

Specifically for volunteers, in Phase III of the study (Table 2), the results unveiled significant disparities in movement execution precision between the two modalities. For slow FE at the inferior limit, the MAE was markedly higher when participants emulated the robot (10.7°), in contrast to the nonrobotic condition (0.4°). This implies that, in this condition, volunteers found it particularly challenging to replicate the precision of NAO's movements. Moreover, even at increased speeds, robot imitation did not seem to confer an accuracy advantage, as indicated by a higher MAE (3.2°) compared to the non‐NAO condition (0.4°). Regarding ER, the scenario was slightly different. At low speeds, participants demonstrated a lower MAE following NAO (16.4°) compared to the session without NAO (22.3°), indicating improved precision. This advantage persists as the speed increases, where MAE values remain lower than in sessions without NAO. In the context of IR, the data did not exhibit substantial differences between the two conditions, suggesting that NAO's presence did not significantly impact the precision of these movements, irrespective of the speed. Regarding the upper limit of Phase III, the presence of NAO facilitated the achievement of ROM within the established limits, as indicated by the zero or lower MAE values compared to the condition without NAO.

The analysis of Phase IV presented a more homogeneous picture (Table 2). In fact, the use of NAO tended to reduce MAE almost for all exercises. In particular, for FE and IR, a lower MAE was observed with the use of NAO for fast speed. For FE, only at a higher speed did the robot allow for the correct execution of the exercise within the limit established in the protocol. Therefore, under certain conditions and for specific movements, robot imitation could actually facilitate a more precise execution. In the context of the ER exercise, the presence of the NAO did not yield a significant enhancement in performance. Notably, the error margin for the higher speed was found to be greater when the robot was utilized.

In the case of patients who only performed exercises of Phase III at a lower speed, results showed that the presence of NAO facilitated the fulfilment of the protocol limits in all movements except for FE at the upper limit (MAE = 4.0°) and ER at the lower limit (MAE = 21.8°), as shown in Table 3.

**Table 3:** MAEs of ROM related to the attainment of the lower and upper FE, ER and IR limits for phase III S_1_ of the patients.

Speed	Exercise	Phase III			
		Inferior limit		Superior limit	
		MAE (°) with NAO	MAE (°) without NAO	MAE (°) with NAO	MAE (°) without NAO
S_1_	FE	0.0	4.9	4.0	1.5
	ER	21.8	18.4	0.0	0.0
	IR	0.0	0.0	0.0	0.0

Abbreviations: ER, external rotation; FE, flexion–extension; IR, Internal rotation; MAE, mean absolute error; ROM, range of motion.

### Time analysis

**Figure 5:**
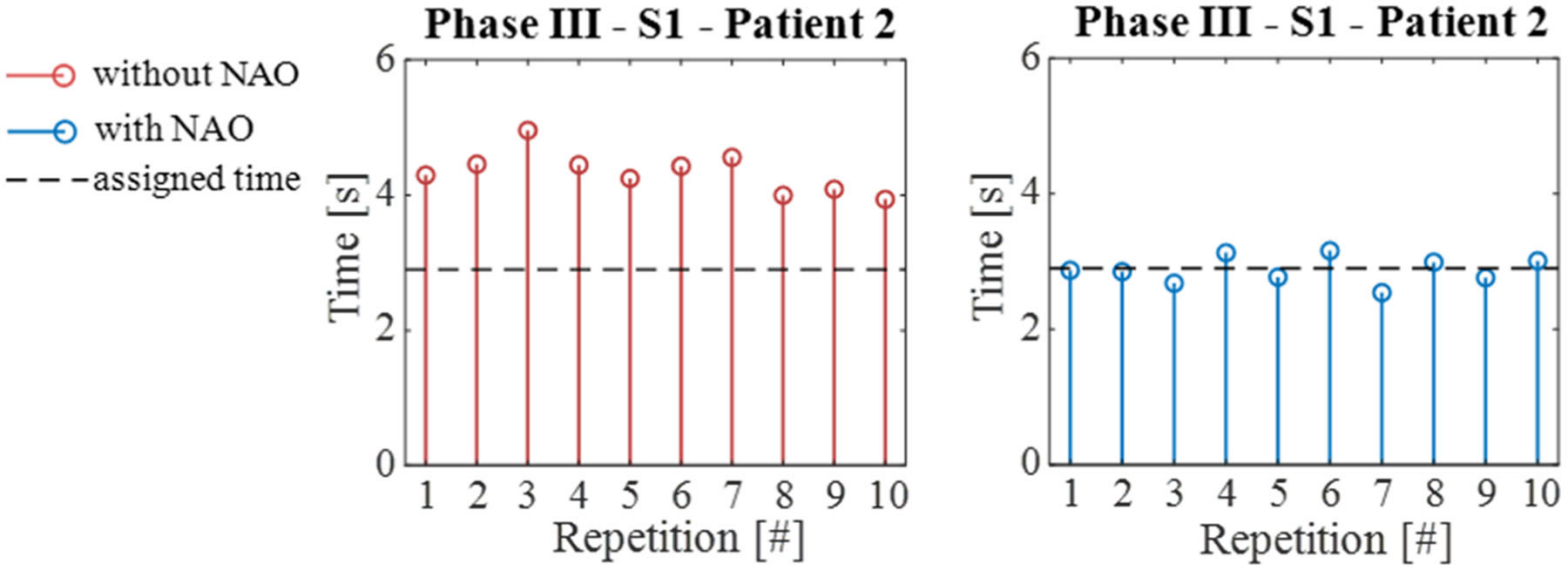
Execution time of each FE repetition performed by patient two during phase III at S_1_ with and without NAO. The assigned time is highlighted by the horizontal dashed line. FE, flexion–extension.

The temporal analysis focused on quantifying the time required by participants to complete each repetition. An example is reported in Figure 5.

**Figure 6:**
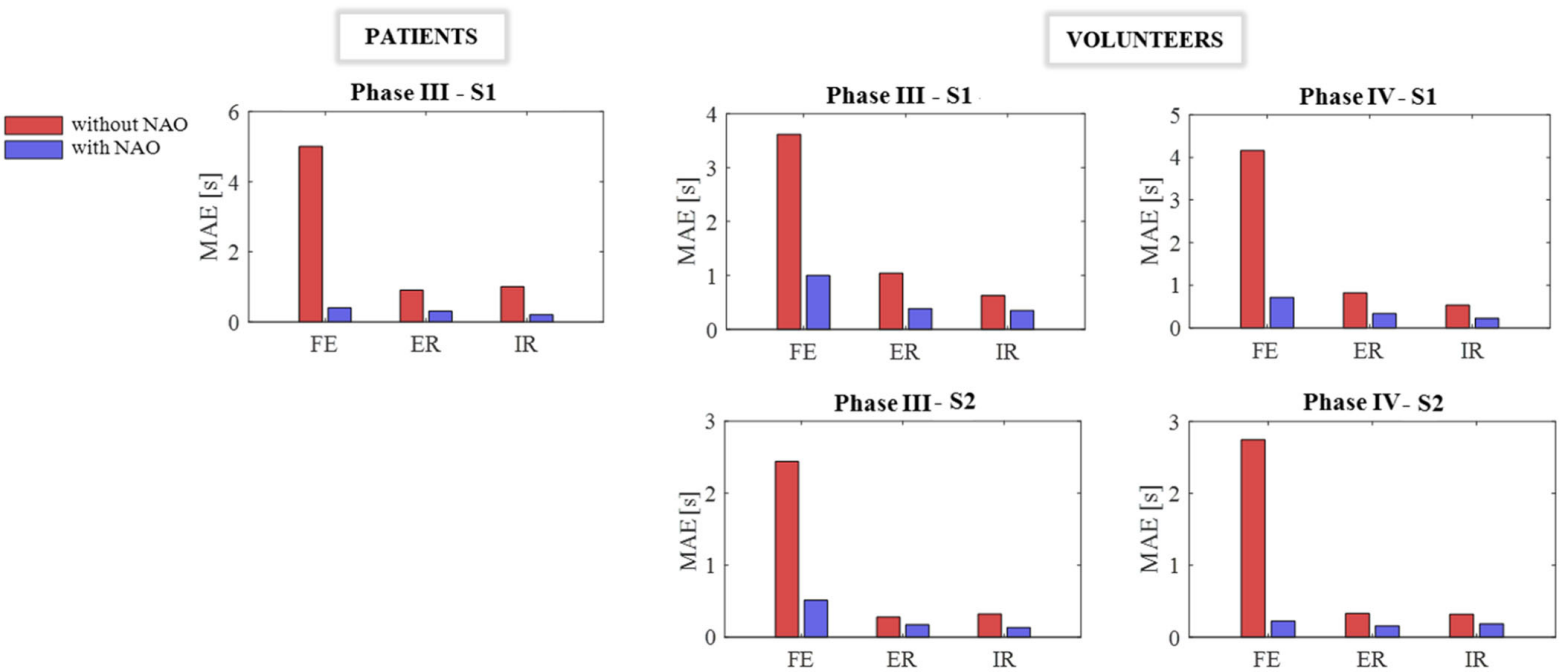
Time MAEs related to patients and volunteers. The MAE value for phase III at speed S1 is given for patients. For volunteers, the MAE values for phase III and phase IV at both speeds, S1 and S2, are given. MAE, mean absolute error.

The analysis of the duration of exercise repetitions (Figure 6) for both volunteers and patients highlights that the use of NAO allows for movements to be performed with a duration that is more consistent with the prescribed assignments. Notably, the time MAE is significantly reduced across all exercises, showing at least a 50% decrease. This reduction is apparent across all phases and speeds, particularly for FE movements.

### Smoothness

**Figure 7:**
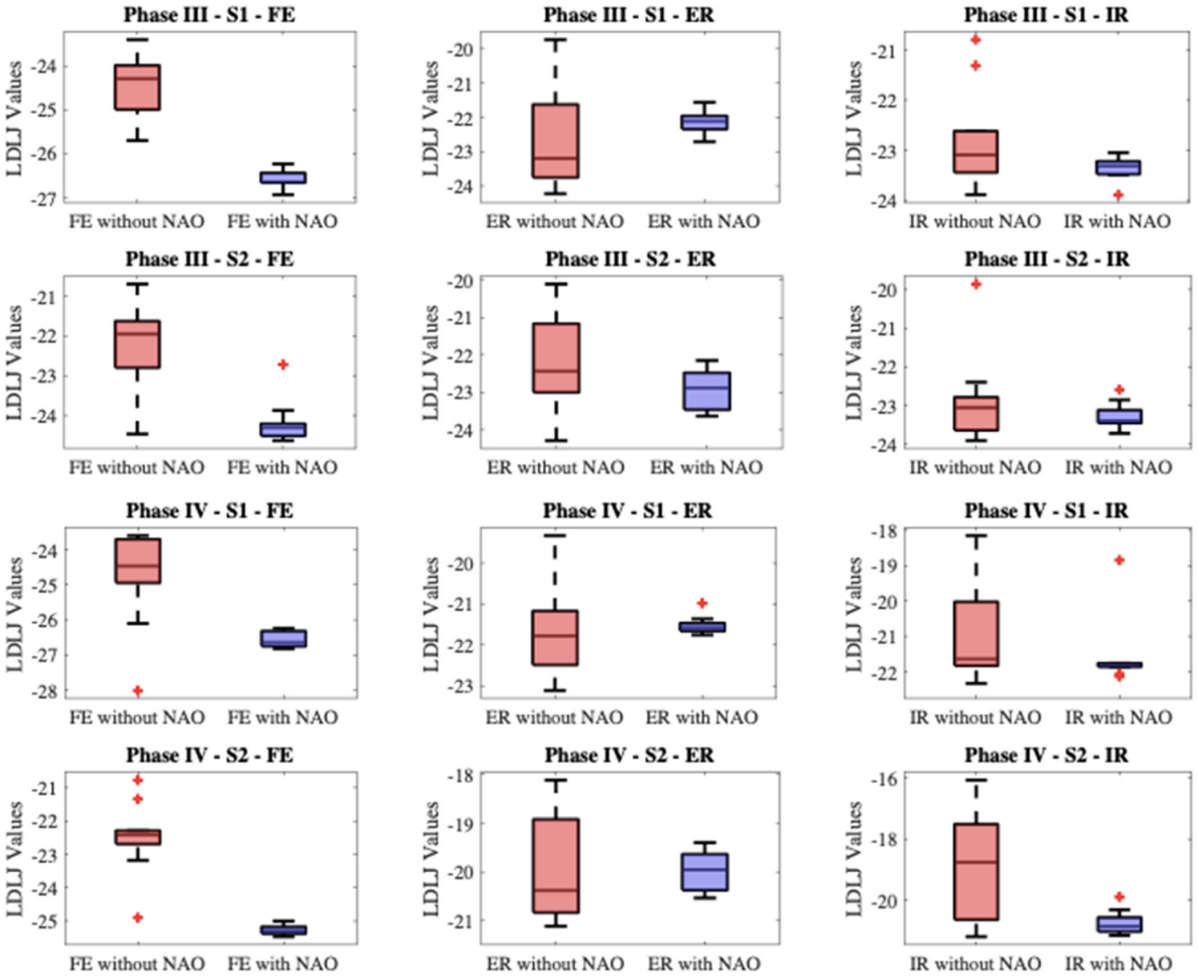
LDLJ values for volunteers are shown by boxplots. ER, external rotation; FE, flexion–extension; IR, Internal rotation; LDLJ, log dimensionless jerk.

Movement smoothness was quantitatively evaluated using the jerk‐based LDLJ metric. A lower LDLJ value signifies smoother movement. The distribution of LDLJ values for the volunteers can be visualized in Figure 7.

In Phase III, where movements were performed at a slower speed S_1_, the support of the NAO robot resulted in a slight reduction in LDLJ values, indicating smoother movements. This beneficial effect becomes more pronounced at the higher speed S_2._ In phase IV, the presence of the robot seems to standardize the quality of movement, reducing the individual variability in jerk (Figure 7). However, patients exhibit greater fluidity in movements with NAO only in FE, as indicated by the lower LDLJ values (see Table 4).

**Table 4:** LDLJ values for patients related to phase III S_1_.

Exercise	LDLJ values	
	With NAO	Without NAO
FE	−27.2	−23.6
ER	−20.8	−21.9
IR	−21.6	−22.3

Abbreviations: ER, external rotation; FE, flexion–extension; IR, Internal rotation; LDLJ, log dimensionless jerk.

## DISCUSSION

This study focuses on the specific application of the NAO robot in shoulder rehabilitation, diverging from the more general upper limb rehabilitation approaches that have predominantly featured in previous research involving NAO in therapeutic settings. Notably, to the best of our knowledge, the factors analysed in this research, including temporospatial control and fluidity of movement, have not been previously evaluated with this approach.

At this preliminary stage, this study has highlighted the primary advantage of employing the NAO robot in rehabilitation sessions, which is its ability to ensure that patients perform exercises within pre‐established time frames by mimicking its movements. Thus, the potential benefit emerging from this study lies in temporal control, which is crucial in the context of rehabilitation [13, 22, 26]. In fact, temporal control involves the capacity to perform movements within specific time intervals, an ability that can be diminished by injuries or medical conditions. To enhance temporal control, it is essential to monitor movement in terms of its initiation and completion.

As previously mentioned, no specific protocol dictates the timing and speed of exercise execution, as these are highly dependent on the patient's condition. Consequently, patients are likely to perform exercises at a pace that aligns with their individual capabilities. Therefore, by defining a temporal reference, embodied by the NAO robot, it can effectively enforce these timings, as evidenced by the significantly lower MAE in timing, even more than 50%, compared to the second session without the robot aid. Indeed, the analysis of the duration of exercise repetitions for both volunteers and patients underlines that the use of NAO brings a temporal execution of movements in line with the predetermined times, dictated by the robot. While various elements, such as a simple video, could serve as a temporal reference, it is important to consider the patient engagement factor. In fact, previous studies have concluded that the physical presence of an entity, particularly an attention‐grabbing humanoid robot‐like NAO, fosters greater patient involvement [21]. Therefore, NAO possesses the capability to conduct personalized therapeutic sessions adapted to the patient's condition, adhering to predetermined timings. Additionally, given this significant benefit, NAO may also reduce the workload for therapists: while the robot cannot replace a therapist, it can certainly support them.

However, there are several areas for improvement, such as revising rehabilitation protocols and the sequence of conducting sessions with and without NAO (since subjects first completed sessions with NAO and then without, which might have influenced the results). Future works will focus on increasing the number of patients enroled in the study to generalize the results and proceed with the validation of the robot‐guided therapy. Expanding the sample size, for example, also with the introduction of a control group, will enable more robust conclusions and enhance the reliability of the findings, providing more definitive insights into the effectiveness of the interventions. Additionally, more rehabilitation sessions are needed to accurately assess the true impact of the robot‐assisted programme. Long‐term studies could further investigate the lasting impact of NAO on overall recovery, including the duration of rehabilitation and post‐rehabilitation quality of life.

## CONCLUSION

NAO showed potential in aiding patients with shoulder musculoskeletal disorders to replicate rehabilitation exercises, guiding both ROM and timing while influencing movement smoothness. Preliminary results demonstrate an improvement in temporal control, which holds significant clinical importance as it is a key factor affecting movement quality. NAO imitation could lead to improved rehabilitation outcomes and enhanced motor learning of motor skills, fostering greater adherence to prescribed therapy.

## AUTHOR CONTRIBUTIONS

The conceptualization and methodology of this study were crafted through collaborative efforts from Alessandra Raso, Arianna Carnevale, Martina Pulcinelli, Alfio Puglisi, Giovanni Pioggia and Emiliano Schena. The development of the software aspects of the study was handled by Alessandra Raso and Alfio Puglisi. Validation was meticulously carried out by Alessandra Raso, Arianna Carnevale and Emiliano Schena, while formal analysis was conducted by Alessandra Raso, Arianna Carnevale, Emiliano Schena, and Umile Giuseppe Longo. Investigative tasks were shared among Alessandra Raso, Arianna Carnevale, Emiliano Schena, and Umile Giuseppe Longo, with essential resources provided by Arianna Carnevale and Umile Giuseppe Longo. Data curation was a joint effort involving Alessandra Raso, Martina Pulcinelli, Arianna Carnevale, and Emiliano Schena, with original draft preparation led by Alessandra Raso, Martina Pulcinelli, Arianna Carnevale, and Emiliano Schena. Review and editing were handled by Alessandra Raso, Martina Pulcinelli, Arianna Carnevale, and Emiliano Schena. Visualization tasks were managed by Alessandra Raso, Martina Pulcinelli, Arianna Carnevale and Emiliano Schena, while supervision was provided by Arianna Carnevale, Emiliano Schena and Umile Giuseppe Longo. All authors have thoroughly reviewed and approved the final manuscript for publication.

## CONFLICT OF INTEREST STATEMENT

The authors declare no conflict of interest.

## ETHICS STATEMENT

This study was approved by the Ethics Committee of Fondazione Policlinico Universitario Campus Bio‐Medico (protocol code: 120/121 OSS ComEt UCBM). Informed consent was obtained from all subjects involved in this study. Informed consent was obtained from all subjects involved in this study.

## Data Availability

Data are available from the corresponding author upon reasonable request.
